# An Insight into Kounis Syndrome: Bridging Clinical Knowledge with Forensic Perspectives

**DOI:** 10.3390/life14010091

**Published:** 2024-01-05

**Authors:** Elena Forzese, Claudia Pitrone, Vincenzo Cianci, Daniela Sapienza, Antonio Ieni, Lorenzo Tornese, Alessio Cianci, Patrizia Gualniera, Alessio Asmundo, Cristina Mondello

**Affiliations:** 1 Department of Biomedical and Dental Sciences and Morphofunctional Imaging, University of Messina, Via Consolare Valeria 1, 98125 Messina, Italy; elena.forzese.94@gmail.com (E.F.); clapitrone@gmail.com (C.P.); daniela.sapienza@unime.it (D.S.); lorenzotornese2013@libero.it (L.T.); patrizia.gualniera@unime.it (P.G.); mondelloc@unime.it (C.M.); 2 Department of Human Pathology in Adult and Developmental Age “Gaetano Barresi”, Section of Pathology, University of Messina, 98125 Messina, Italy; aieni@unime.it; 3 Department of Cardiovascular Medicine, Fondazione Policlinico Universitario A. Gemelli—IRCCS, Largo A. Gemelli 8, 00168 Rome, Italy; alessiocianci.1998@gmail.com

**Keywords:** Kounis syndrome, anaphylaxis, postmortem, immunohistochemistry, biochemistry, tryptase, acute coronary syndrome

## Abstract

Kounis syndrome (KS) is an acute coronary syndrome triggered by allergic or hypersensitivity reactions. Incidence rates vary, with studies reporting 19.4 per 100.000 among all admissions and 3.4% among allergy patients. This review explores the expanding understanding of KS, encompassing various manifestations, and focusing on both clinical data and forensic findings useful in performing a diagnosis. The pathophysiology of this syndrome involves a complex interplay between allergic reactions and the cardiovascular system. Mast cell activation, histamine release, leukotrienes, cytokines, and platelet activation can contribute to coronary events. Three types of classification systems (allergic angina, allergic myocardial infarction, allergic stent thrombosis) aid in categorizing presentations. The diagnosis of KS relies on clinical presentation, laboratory findings, and imaging. Postmortem assessment of KS is based on the integration of circumstantial data, autopsy, and histological findings. Biochemical and immunohistochemical analyses also contribute to postmortem diagnosis. In conclusion, a combined, multidisciplinary approach should be used to ease the diagnostic process, which is crucial for forensic practitioners in confirming KS occurrence.

## 1. Introduction

Kounis syndrome (KS) is an acute coronary syndrome that occurs in the setting of allergic or hypersensitivity reactions, first described by Nicholas Kounis [1,2]. It encompasses a spectrum of allergic reactions leading to acute coronary events. The mechanism involves the release of inflammatory mediators during an allergic response, which can trigger coronary artery spasm or plaque rupture, leading to angina or myocardial infarction. The first two cases were presented by Kounis and Zavras [1], who reported symptoms of angina-like chest pain and electrocardiographic changes during allergic reactions. The symptoms were initially attributed to coronary artery spasm triggered by an allergic response. Since its first discovery, more and more cases have been reported in the medical literature. The disease mechanisms have been further analyzed, expanding the definition of KS to not only a single-organ disease but a complex multisystem disease [3], which includes any coronary syndrome related to mast-cell-associated disorders and inflammatory cell interactions, such as allergic angina, allergic myocardial infarction, and allergic stent thrombosis, as well as all their implications. Moreover, some work has been done to generate a classification and subdivision of the disease in three subtypes to facilitate diagnosis and treatment [4,5,6].

As depicted in a recent study [7], the interplay between anaphylaxis and KS is quite complex, and it can involve the skin, respiratory, neurological, and cardiovascular systems. This significantly influences both morbidity and mortality, calling for larger population studies, case reports, and appropriate management plans.

The aim of this review is to analyze the epidemiological and pathophysiological aspects of KS, both to provide an insight into the disease and to describe the main literature data regarding the forensic tools used for an appropriate postmortem assessment.

## 2. Epidemiology and Pathophysiology

KS is considered a relatively rare condition, and its true prevalence is not well established due to under-recognition and under-reporting [6].

Recent reports suggest that KS has been observed across all races, age groups (from 2 to 90 years old), and geographic locations. It has mostly been reported in Southern Europe, particularly in Turkey, Greece, Italy, and Spain [6]. This geographical variation may be attributable to physician awareness, climate, environmental conditions, overconsumption of medicines, or inadequate preventative measures. An example is the district of Achaia, Greece, where 52 cases were reported in the last 4 years, resulting in an estimated annual incidence of 4.33 cases per 100,000 inhabitants [6].

In a prospective study lead by Akoz et al. [8], it was found that out of 138.911 patients admitted to the emergency department in one year, 793 presented with allergy complaints. The incidence of KS among all admissions and allergy patients was 19.4 per 100,000 (27/138.911) and 3.4% (27/793), respectively.

Between 2010 and 2014, 51 cases of KS were reported to the International Pharmacovigilance Agency, with almost half occurring in 2014 [9].

In a retrospective study lead in the Swiss Canton [10], the incidence of anaphylaxis with circulatory symptoms was estimated over a 3-year period, revealing 246 episodes in 226 individuals, with an incidence of 7.9–9.6 per 100,000 inhabitants per year. The case–fatality rate was 0.0001%, with three reported deaths.

**Figure 1:**
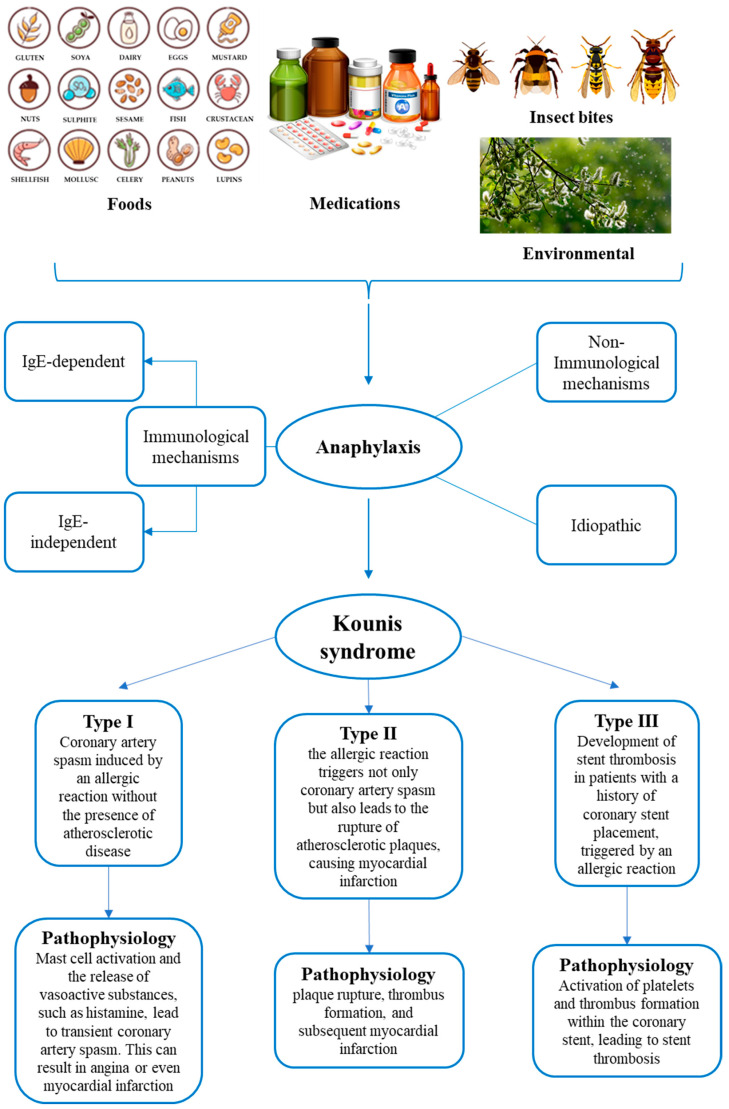
Summary of the main triggering factors of anaphylaxis, pathophysiological mechanisms of anaphylaxis, and types of Kounis syndrome.

Various causes can trigger KS, such as foods, medications (i.e., antibiotics, nonsteroidal anti-inflammatory drugs, contrast media), and environmental factors, including insect bites [6,9,10] (Figure 1). It is also important to underline how some antiblastic drugs, such as those used for molecular target therapy and immune checkpoint inhibitors, are considered capable of inducing vasospastic angina too [11].

The pathophysiology of KS involves a complex interplay between allergic or hypersensitivity reactions and the cardiovascular system. The pathophysiological mechanisms of anaphylaxis can be distinguished as immunological (IgE-dependent and IgE-independent, respectively), non-immunological, and idiopathic [12]. The main molecular mechanism is the allergic IgE-mediated reaction, which involves mast cells and basophils, responsible for the release of inflammatory mediators in systemic circulation. The non-IgE-mediated reaction, called anaphylactoid, is based on IgG and IgM immune complexes activation. Then, there are cases in which a non-immunological mechanism is described, where a direct degranulation of mast cells and basophils occurs. Finally, in idiopathic anaphylaxis, no triggering causes can be identified.

The most reported pathophysiological mechanisms of KS consist of coronary vasospasm, platelet activation, aggregation, and platelet-dependent arterial smooth cell hyperplasia [13], determined by allergic inflammatory mediators. Particularly, alterations in sympathetic and parasympathetic neural transmission, local arterial metabolic abnormalities, extensive responsiveness of the medium-sized arteries to circulatory vasoactive amines, or a combination of these factors have been described as potentially responsible for the onset of coronary spasm [14]. Moreover, the presence of mast cells in the culprit lesion, especially if complicated by plaque erosion or rupture, can be identified in acute coronary syndrome due to allergic events [15]. Mast cells activation is responsible for the degranulation of vasoactive mediators (i.e., histamine, chemokine, tryptase, chymase, cathepsin-D, heparin, proteoglycans, and cytokines), which are then released into local and peripheral circulation, determining vasoconstriction [16]. Among these mediators, histamine induces both coronary vasoconstriction and platelet activation, with coronary thrombosis [17]. Tryptase contributes to coagulation cascade activation, promoting thrombosis and fibrinolysis [18]. Then, the activation of metalloproteinases promoted by tryptase and chymase acts as trigger for collagen degradation, inducing the destabilization of pre-existing atherosclerotic plaques, as well as their erosion and rupture [19]. Evidence from the literature highlights that in KS, it is possible to identify both indirect and direct heart involvement [15]. In the first case, hemodynamic alterations, consisting of severe vasodilatation due to inflammatory mediators, lead to distributive shock and then to myocardial disfunction. In latter cases, it is possible to describe direct cardiac involvement, characterized by coronary vasospasm or thrombosis, myocardial ischemia/infarction, up to acute heart failure. A direct cardiac effect could manifest also with myocardial dysfunction related to the direct action of some mediators, capable of myocardial activity suppression.

KS can manifest in various ways, ranging from angina to acute myocardial infarction. The specific presentation may depend on factors such as the type and severity of the allergic reaction, individual patient characteristics, and the presence of pre-existing cardiovascular conditions [6].

Understanding the pathophysiology of KS is crucial for appropriate diagnosis and management. Treatment often involves addressing the underlying allergic reaction, managing coronary artery spasm, and providing supportive care for cardiovascular events. To facilitate this, a classification system for KS has been provided [4,6]. Of the three existent types, each one presents different pathophysiology characteristics and clinical assessment, but there can be an overlap between the types, so individual cases may not fit into one specific category. It is important to note that these classifications provide a framework for understanding the different clinical scenarios associated with KS [6,20]. Type I variant, also known as vasospastic allergic angina or MINOCA (myocardial infarction with nonobstructive coronary arteries) type, usually develops in subjects with no atherosclerotic phenomena in coronary arteries and with no other risk factors, manifesting itself with endothelial dysfunction or microvascular angina: the releasing of the inflammatory mediators typical of anaphylaxis could cause coronary spasm with no cardiac enzyme alterations or myocardial infarction, leading to death in the most severe cases [6,20]. The type II variant is instead an allergic acute myocardial ischemia which develops in subjects suffering from coronary atherosclerosis: the release of inflammatory mediators can determine transient events characterized by coronary spasms with normal cardiac enzymes up to the rupture of plaques and death [6,20]. Lastly, the type III variant occurs in patients with coronary stents, at which occur thrombotic phenomena secondary to the onset of the anaphylactic phlogistic phenomena. There are two subtypes in the Type III variant, stent thrombosis (subtype IIIa) and/or stent restenosis (subtype IIIb). In these cases, it is possible to detect eosinophils and mast cells on histological examinations performed after autopsy [6,20]. The three main types are reported in Figure 1. These classifications help in understanding the diverse ways in which the syndrome can manifest and, therefore, be treated.

## 3. Clinical Presentation

KS is a complex multisystem disease related to allergy-hypersensitivity that affects the skin and respiratory and cardiovascular systems, involving not only the coronary arteries but also the cerebral and mesenteric ones [3,6]. As there are no specific findings of KS, the evaluation of the patient’s clinical history can help to identify the etiological and clinical correlation between any allergic triggering factors and the onset of acute coronary syndrome [20]. Risk factors include atopic susceptibility with previous allergic episodes, hypertension, smoking, diabetes, and hyperlipidemia [21].

Clinically, KS manifests as a combination of an acute, subacute, or chronic allergic reaction (usually anaphylaxis) simultaneously associated with cardiac symptoms, including chest pain, dyspnea, chest discomfort, palpitation, tachycardia or bradycardia, pulmonary oedema, coronary vasospasm, angina pectoris, myocardial infarction, and acute heart failure [21,22]. In most cases (80%), symptoms appear within an hour of exposure to the allergen, although in 9,2%, later onset is possible after more than 6 h [21]. Regarding cardiac involvement, the most common symptoms and signs are chest pain (60%) and hypotension (75%) due to distributive shock, related to extensive peripheral vasodilation, and cardiogenic shock with the suppression of myocardial function [15,23]; it is also possible to observe dermatological (70%), respiratory (30%), and gastrointestinal (20%) symptoms [22,23]. Dermatological manifestations, such as urticaria, rash, erythema, and angioedema, representing classic findings in the case of allergic reaction/anaphylaxis can be useful for the diagnosis of KS, although they may be absent or delayed. However, the absence of cutaneous manifestations is not a criterion for excluding KS but rather a sign of severe cardiogenic and distributive shock; during anaphylaxis, cardiac collapse, determining a marked reduction in cardiac output with vasoconstriction and hypotension, can drastically reduce venous return and delay the action of anaphylactic mediators at the skin level [24]. Common symptoms and signs related to allergic/anaphylactic reaction and in KS also include general malaise, nausea, vomiting, fainting, diaphoresis, cold extremities, paleness, and palpitations [22,24].

Furthermore, reduced cerebral perfusion related to an anaphylactic reaction can cause neurological symptoms such as headache, tiredness, drowsiness, or altered neurological status, which are frequently considered not to be noteworthy because of their poor specificity, and so lead to KS misdiagnosis. [24]. The consequences of post-allergic coronary syndrome, or cardiac anaphylaxis, may be myocardial ischemia, acute myocardial infarction, conduction defects, malignant arrhythmias (i.e., ventricular fibrillation), cardiac muscle cell dysfunction, and, in the absence of treatment, cardiorespiratory arrest or sudden cardiac death [20].

## 4. Clinical Diagnosis

The diagnosis of KS is based on clinical presentation, laboratory findings, ECG, echocardiography, and coronary angiogram [3,6,7,21,24]. In patients with symptoms and/or signs of systemic allergic reactions, especially if associated with cardiac symptoms, it is necessary to examine electrocardiographic findings and measure troponin levels to exclude or confirm the diagnosis of KS [6,21].

Measuring serum tryptase, IgE antibodies, histamine, cardiac enzymes (i.e., CK and CK-MB), and cardiac troponins is particularly helpful to better define the diagnosis of KS [24]. Serum tryptase represents an indicator of mast cell activation [25], and its levels begin to increase only 30 min after the onset of the signs and symptoms of allergic reaction, reaching their peak during the following 2 h [3,6,24]. In clinical practice, the value of serum tryptase concentration indicating anaphylaxis must be higher than 11.4 ng/mL, which has a sensitivity of 73% and a specificity of 98%; furthermore, due to the short half-life (about 90 min) [6,23], serial dosing with a minimum of three determinations is recommended [20]. Histamine is rapidly released from mast cells and remains in circulation for only about 8 min after an allergic event; thus, it is recommended that blood samples are collected immediately after the onset of chest pain [3,6]. Cardiac enzymes are directly correlated to the severity of the anaphylactic reaction, and their increase is a marker of cardiac damage [20]. Particularly, the increase in CK and CK-MB and cardiac troponins support the main involvement of both coronary arteries and/or the heart during anaphylaxis [3,22,24]. Indeed, in the case of anaphylaxis, angioedema, urticaria, and urticaria–angioedema, troponin I levels are higher compared to either healthy patients [22,24] or those with milder allergic reactions [3]. For this reason, these enzymes should be dosed to promptly identify heart damage attributable to KS to provide quick management [6].

The acute release of inflammatory mediators in type I can provoke coronary artery spasm without an increase in cardiac enzymes or can determine coronary artery spasm up to acute myocardial infarction with raised cardiac biomarkers. Similar findings are described for type II, in which it is possible to detect either coronary spasm associated with normal levels of cardiac biomarkers or signs of plaque erosion or rupture responsible for acute myocardial infarction, determining the increase in cardiac biomarkers [3,23].

KS diagnosis is performed also by electrocardiogram (ECG) echocardiogram and a coronary angiography [3,23].

ECG in KS shows as the most common finding the elevation of the ST segment in the anterior and inferior leads, related to vasospasm of the right coronary artery, which is the most frequent vessel involved. It is also possible to detect findings of heart block and cardiac arrhythmias of any degree. In patients affected by type I KS, in which cardiac enzymes and coronary angiography may be normal and any electrocardiographic abnormalities are transient, it is essential that ECG monitoring is repeated several times to identify KS early and promptly [20,23,24]. Cases may also occur with normal or non-specific ECG; if no elevation or depression (existing or transient) of the ST segment, no inversion of the T wave, and flat or pseudo-normalization of T waves are detected, reperfusion surgery may be delayed [23].

Echocardiography and coronary angiography allow for the detection of cardiac wall abnormalities and define the anatomy of coronary arteries [6]. The echocardiogram integrates the information given by the ECG, providing data about the abnormal movement of the cardiac wall normally supplied by the coronary artery involved in the disease [23]. Angiography is the main investigation used for the assessment of the cause of coronary abnormality because it can establish whether the obstruction to coronary flow is due to vasospasm or thrombosis, thus representing a useful tool for the differential diagnosis between the three KS variants [20,22].

Newer techniques such as single photon emission computed tomography (SPECT) with thallium-201 and SPECT with 125I-15-(p-iodophenyl)-3-(R, S) methylpentadecanoic acid (BMIPP), in conjunction with cardiac magnetic resonance imaging, are useful to better define the actual cardiac damage, especially in the type I variant of KS when the coronary angiography is found to be normal [3,7]. Cardiac SPECT and cardiac MRI have proven effective both for revealing severe myocardial ischemia and subendothelial damage, and for distinguishing KS from myocarditis (although the differential diagnosis is histological) [22,24]. In dynamic cardiac magnetic resonance imaging (MRI), delayed contrast-enhanced images show normal washout in the subendocardial lesion in patients with variant KS type I [6,7].

## 5. Postmortem Assessment of Kounis Syndrome

The postmortem assessment of anaphylactic death is considered a challenge because the evidence emerging from autopsy and histology is not pathognomonic. The diagnosis should be based on the integration of circumstantial and anamnestic data, autopsies, histological findings, and biochemical and immunohistochemical data [26,27]. This approach has a pivotal role also in those cases where the occurrence of KS is suspected after analyzing the anaphylaxis effectors in the biological fluids and in common target tissues, together with heart and coronary arteries [28].

Anaphylactic deaths are often related to a combination of factors, including upper airway obstruction from mucosal edema, asphyxia from bronchospasm, and shock due to massive fluid shifts [26]. Thus, at postmortem evaluation, the main gross findings can be observed in the respiratory system, where the presence of laryngeal edema, tracheo-bronchial hypersecretion of mucus and mucosal edema with lumen obstruction, and pulmonary edema can support the occurrence of anaphylaxis [27]. Histological analysis provides the confirmation of these macroscopic data and, also, can show other signs such as bronchospasm, emphysema, and acute pulmonary edema [26]; then, an important finding, strongly related to anaphylaxis, is leukocyte infiltration, mostly mediated by basophils, eosinophils, and mast cells [23].

In cases with KS occurrence suspicion, histological examination of the heart and coronary arteries is strongly recommended. For this purpose, the use of different histological stains can highlight the presence of different inflammatory cells involved in allergic reactions. In fact, eosinophils and mast cell infiltrates are identified using hematoxylin–eosin and Giemsa (or toluidine blue) staining, respectively [29,30].

In KS manifesting as coronary spasm, a high presence of mast cells has been found in the coronary arteries wall, including the area of the spasm, especially in the tunica adventitia [29,30,31,32,33]. Furthermore, many studies report that the major count of mast cells is concentrated in atherosclerotic and hemorrhagic plaques [28,29]. Histologically, myocardial alterations caused by the ischemic insult related to the compromission of coronaries due to heart anaphylactic involvement can be observed. In fact, when death occurs within a short period of time from ischemia onset, it is possible to find signs such as myofiber eosinophilia, elongation of sarcomeres and nuclei, wavy fibers, interstitial oedema, and contraction band, suggesting the occurrence of a so-called early myocardial ischemia [34,35]. Kitulwatte et al., reported the presence of transmural contraction band necrosis together with consistent inflammatory cell infiltrates [27].

However, since these findings cannot be considered pathognomonic, other analyses, such as biochemistry and immunohistochemistry, can contribute to performing the postmortem diagnosis of anaphylaxis and KS [26].

Biochemical investigations are described in many studies as a useful tool for the postmortem assessment of anaphylaxis, with a particular attention paid to the measurement of serum tryptase and IgE. Serum tryptase is the most used biomarker of anaphylaxis and is a very stable enzyme, being detectable up to 6 days after death [26,28]. However, since postmortem degradation processes can lead to a reduction in the effective concentration of tryptase as the postmortem interval (PMI) increases, in case of suspected anaphylactic death, it is suggested that a blood sample is taken as soon as possible [26]. Various cut-offs for serum tryptase levels from peripheral blood have been reported in the forensic literature. As reported by Kounis et al. [28], a level of tryptase of 10 µg/L or greater has a sensitivity of 86% and specificity of 88% for the diagnosis of postmortem anaphylaxis. Tse et al. and Edston et al. [36,37] proposed a tryptase cut-off value ≥ 53.8 µg/L and 45 µg/L on femoral blood, respectively. The latter one can be considered to be a new limit value [36,37]. However, if blood is collected from central vessels, such as the aorta, the suggested threshold value is 110 µg/L [38], which is higher than previous ones, since some factors, such as prolonged cardiac massage or defibrillation, are responsible for an increase in mast cell degranulation and in tryptase levels due to visceral trauma from chest compressions [39]. Therefore, peripheral blood is preferable to central blood for postmortem tryptase determination [40,41]. However, even in the case of peripheral blood, in forensic practice, it is necessary to highlight that some factors can influence the tryptase concentration (i.e., hemolysis and duration of the agonal period). Furthermore, increased tryptase levels have also been described in non-anaphylactic deaths [27,28], such as sudden infant death syndrome, acute deaths after heroin injection, traumatic deaths, and asphyxia.

A total and specific IgE assay in postmortem serum can provide data on anaphylaxis occurrence, demonstrating atopic disposition and the degree of sensitization to a particular allergen [26,27]. Some studies argue that serum IgE measurement provides approximate data due to the short half-life and lack of a cut-off value for the differential diagnosis of anaphylaxis from other allergic diseases (i.e., asthma) [42]. Nevertheless, integrating the results of these analyzes with serum tryptase levels can still provide useful information on the cause of death.

In suspicion of KS, the evaluation of these serum analytes should be performed and integrated thorough the analysis of others anaphylaxis-related substances (such as carboxypeptidase A and histamine) and specific IgE in the pericardial fluid. Specifically, an increased concentration of carboxypeptidase A, secreted by mast cells, has been found in both postmortem serum and the pericardial fluid of subjects who died from anaphylaxis [28]. 

Moreover, since KS can cause coronary vasospasm or coronary thrombosis up to early myocardial ischemia or infarction, a further useful biochemical investigation consists of the measurement of serum troponin I levels to evaluate cardiac damage and to support its postmortem diagnosis [43].

Immunohistochemistry is another useful investigation for a more effective postmortem diagnosis of anaphylaxis, although it is important to underline that the identification of mast cells in tissues cannot be considered sufficient to make a diagnosis of certainty. Indeed, an increased number of mast cells can be detected also in various biological processes (i.e., tissue remodeling, angiogenesis, fibrosis, and asphyxia) and in non-anaphylactic deaths. The main effectors of inflammation during anaphylaxis (i.e., eosinophils, basophils, but above all mast cells) have been found in tissues, as bronchial, respiratory, and intestinal mucosa, red pulp of the spleen, and connective tissue (i.e., cutaneous and perivascular) [26,30,33].

A research group immunohistochemically analyzed eosinophil and basophil infiltrations using, respectively, major basic protein (MBP) and proMBP1 antibodies [44]. These markers may also be used to detect specific cell infiltration in myocardial tissue to better define heart anaphylactic involvement and then to support KS occurrence.

Mast cells are the main effectors of anaphylaxis and are frequently immunohistochemically detectable in many tissues, such as respiratory and spleen, using anti-tryptase antibodies [42]. These inflammatory cells are a very useful KS assessment, having been observed in myocardial tissue and coronary arteries [28]. Increased numbers of mast cells in the three layers of the coronary arteries and myocardial cellular infiltrates of neutrophils with mast cells and eosinophils have been detected in patients who died because of coronary spasm. Therefore, during anaphylaxis, pericardial tissue may also be involved, in which histological examination has revealed perivascular areas of infiltration of lymphocytes, macrophages, neutrophils, and mast cells [7,28]. These findings suggest that, in KS, myocardial damage seems to be related to the effect of both mast cell degranulation and the release of inflammatory mediators that affect the cardiovascular system (i.e., coronary vasoconstriction induced by histamine). Thus, a diagnosis should be made mainly using the immunohistochemical evaluation of degranulated tryptase in coronary walls and myocardial tissue [26,38,45,46,47,48]. Moreover, some studies also reported the analysis of chymase, representing another marker for mast cells detection [38,48]. In particular, Del Duca et al., reported that in some samples, it is possible to identify anti-chymase positive mast cells in perivascular spaces [49]. Therefore, these data could be useful for an even more correct identification of KS. A further diagnostic method is represented by anti-CD117, a specific indicator of the activation of the cKIT receptor tyrosine kinase, required for mast cell maturation. Therefore, anti-CD117 may be used to detect the activated tissue mast cells also in myocardial tissue and coronaries [45,46,47].

**Figure 2:**
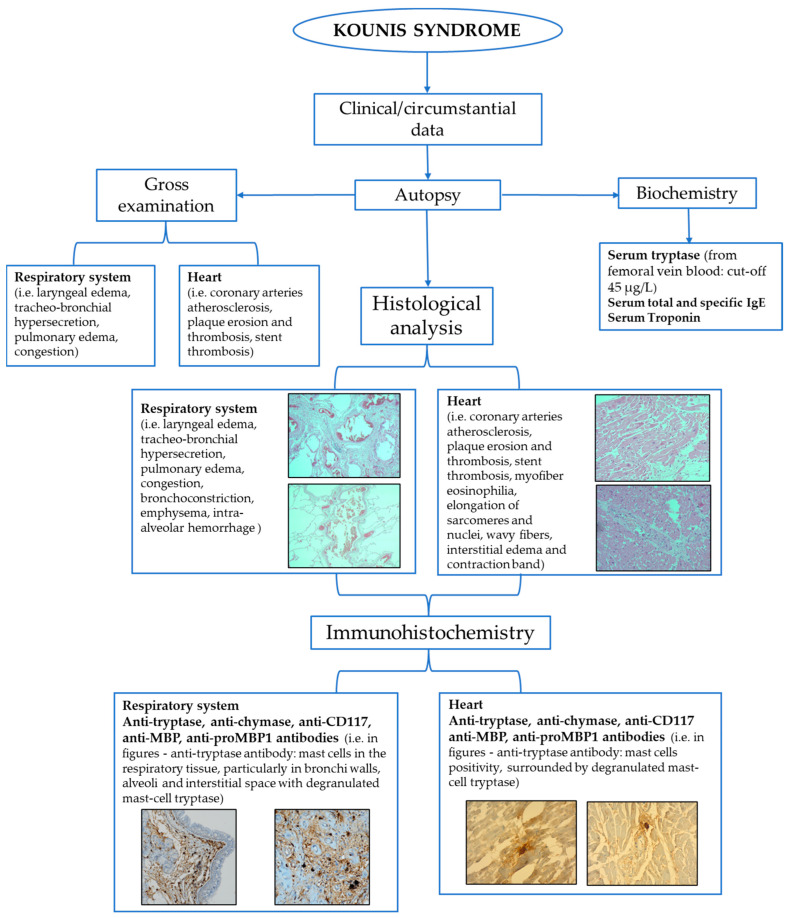
Flow-chart of the main tools for postmortem assessment of Kounis syndrome.

A schematic summary of the main investigations to reach the postmortem diagnosis of Kounis syndrome is reported in Figure 2.

## 6. Limitations

The literature analysis revealed that most of our knowledge regarding KS derives from case reports and small case series, in which the disease has been diagnosed by both clinical presentation and instrumental findings of acute coronary syndrome. Consequently, limited knowledge has been found about KS syndrome pathogenesis. The pathophysiological mechanisms to date, which can be synthetically distinguished in those with a direct or indirect cardiac involvement, have only been hypothesized, not completely clarified: among these, the potential predisposing role of genetic and molecular factors, which could influence the development of the syndrome, has not yet been sufficiently investigated. Anyway, in the forensic field, the diagnosis of anaphylactic death can be obtained by the integration of circumstantial and anamnestic data, autopsy, histological, immunohistochemical, and biochemical findings [45]. This methodological approach also serves to define KS occurrence. However, the absence of circumstantial/anamnestic and macroscopic data, considered a “guide” for setting up postmortem analysis, may represent an important limitation in the diagnosis of the syndrome, not being in this case executed by biochemical and immunohistochemical investigations (not routinely performed) useful for KS assessment.

Another limitation can be related to the postmortem interval (PMI). In fact, the increase in PMI leads to the onset of postmortem autolytic and transformative phenomena, with the consequent alteration of histological, immunohistochemical, and biochemical findings. Finally, it is necessary to underline that immunohistochemistry represents a semi-quantitative investigation and currently in the forensic field, there are no shared minimum values of immunopositivity leading to a certain diagnosis.

## 7. Conclusions

Kounis syndrome, also known as allergic myocardial infarction or allergic angina syndrome, is characterized by the combination of an allergic/anaphylactic reaction, leading to the release of specific inflammatory mediators and the onset of an acute coronary syndrome, with ischemic myocardial damage. Even if the clinical diagnosis of KS is already established using several laboratory and instrumental investigations, postmortem diagnosis is a challenge for the pathologist.

KS assessment in the forensic setting requires the integration of clinical/anamnestic data, with the findings belonging to multiple investigations. Firstly, macroscopic examination of the heart provides information on the coronary arteries; histological examination of myocardium and coronary arteries can reveal inflammatory infiltrates, such as mast cells and eosinophils. Biochemical and immunohistochemical analyses may offer significant data. Biochemical investigation for inflammatory mediators (i.e., histamine, tryptase, total, and specific IgE) can reveal increased values suggesting an anaphylactic reaction; on the other hand, troponin I represents a marker for acute myocardial damage. Then, immunohistochemistry integrates previous investigations to ascertain the mast cell infiltrate and, above all, the presence of degranulated tryptase at coronary arteries supporting KS occurrence. Furthermore, in addition to the evaluation of tryptase, the evaluation of chymases and CD 117 could be useful.

In conclusion, even if forensic evidence lacks a standardized diagnostic model to assess KS occurrence, the implementation of a multiparametric approach can help the pathologist in forensic practice.

## Data Availability

All data are reported in the manuscript.
